# The First Case Report of X-Linked Sideroblastic Anemia With Ataxia of Chinese Origin and Literature Review

**DOI:** 10.3389/fped.2021.692459

**Published:** 2021-07-20

**Authors:** Shiqiu Xiong, Yang Jia, Shijun Li, Peng Huang, Jie Xiong, Dingan Mao, Qingnan He, Liqun Liu

**Affiliations:** ^1^Department of Pediatrics, The Second Xiangya Hospital, Central South University, Changsha, China; ^2^Children's Brain Development and Brain Injury Research Office, The Second Xiangya Hospital, Central South University, Changsha, China

**Keywords:** X-linked sideroblastic anemia with ataxia, XLSA/A, sideroblastic anemia, ataxia, ABCB7 gene

## Abstract

X-linked sideroblastic anemia with ataxia (XLSA/A) is a rare X-liked inherited disease, which was linked to the ABCB7 gene mutations. So far, five families have been reported worldwide. We present the first Chinese family of XLSA/A with novel ABCB7 gene mutation (c.2024A > G) and make a retrospective literature review. All affected patients were male. Age of symptom onset was <2 years old. The main symptoms included ataxia, delay in motor development, and mild sideroblastic anemia with obviously increased erythrocyte protoporphyrin. In this case, he had new symptoms that had not been reported in other cases such as epilepsy and cryptorchidism. We also discuss the possible molecular mechanism linking ABCB7 gene mutations to sideroblastic anemia and ataxia.

## Introduction

X-linked sideroblastic anemia with ataxia (XLSA/A) is a rare X-linked inherited disease, which is linked to the ABCB7 gene mutation on the long arm of the X chromosome at Xq13 (1, 2). XLSA/A is characterized by early-onset ataxia and usually mild sideroblastic anemia (3). Growth retardation and strabismus are also common symptoms (3–6).

The ABCB7 gene encodes a mitochondrial adenosine triphosphate (ATP)-binding cassette (ABC) transporter protein (ABCB7), which locates in the inner membrane of the mitochondria (3, 7). ABCB7 could export sulfur-containing compound from the mitochondria to cytosol (4). The present study has demonstrated the involvement of ABCB7 in the biosynthesis of heme via interaction with ferrochelatase and ABCB10 (8, 9).

Here, we present the sixth identification of XLSA/A, which is the first case in China. We also review the previous literatures and discuss the symptoms and auxiliary inspection findings of XLSA/A.

### Case Presentation

The proband was a 5-year-old boy who was admitted to our hospital for recurrent seizure and growth retardation for 4 years. He was born at 35 weeks with a birth weight of 2,000 g. He had nystagmus at the first month of age. Growth and motor delays were noted at early childhood. He can sit at 1 year of age. He was unable to walk unsupported while still rapidly losing his balance after taking a few steps at around 2 years of age. At the age of four, he can walk and speak a few words.

The patient had a history of anemia. His mother was diagnosed mild anemia at 38 years old but did not receive any treatment; his mother's sister was also diagnosed with mild anemia. His grandmother's brother had intellectual problem and died at an early age with unknown cause, and his grandfather died because of respiratory disease without any neurological symptoms.

The patient's first generalized seizure occurred at 11 months of age. He initially failed treatment with a single dose of levetiracetam (40 mg/kg/day) but achieved seizure control with a combined therapy of levetiracetam (40 mg/kg/day) and topiramate (1.5 mg/kg/day). Gesell Development Scales (GDS) estimated at 5 years old indicated severe to extremely severe delay (scores of Gesell gross motor, fine motor, visual motor, language, and social were 28, 37, 31, 24, and 30, respectively). Scores of the Checklist for Autism in Toddlers-23 (CHAT-23) and the Clancy Autism Behavior Scale (CABS) were 20 and 15, respectively, both of which indicated he might have autism.

**Figure 1:**
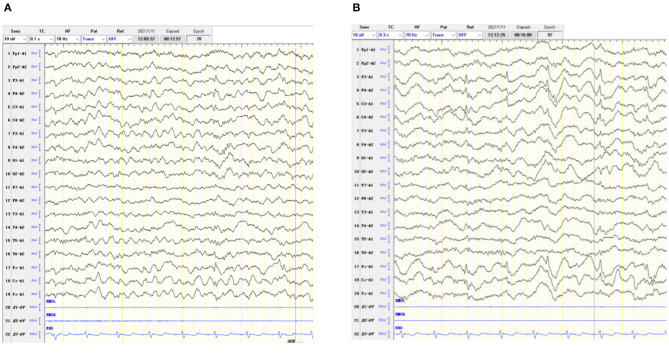
Interictal electroencephalogram was found with spikes and sharp waves over the bilateral frontal region, anterior midline region and centrotemporal region during both the waking **(A)** and sleeping **(B)** periods.

**Figure 2:**
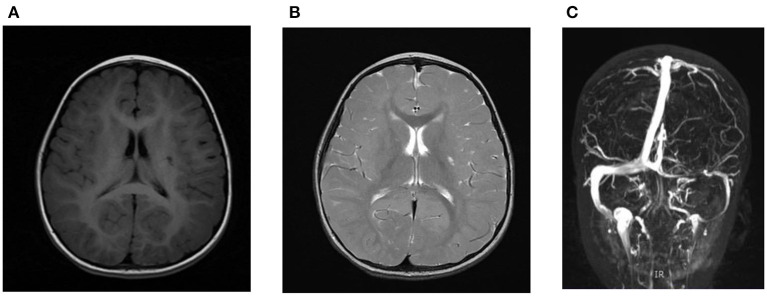
MRI showed lacunar infarction in the left basal ganglia with glial proliferation around in T1-flair **(A)** and T2-fse imaging **(B)**; MRV showed small tiny left transverse sinus, sigmoid sinus, and internal jugular vein **(C)**.

**Figure 3:**
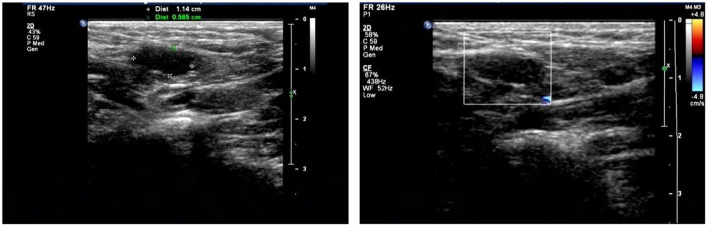
Ultrasonography showed bilateral cryptorchidism.

At the age of 5 years and 4 months, his height (97.5 cm), weight (15 kg), and head circumference (44 cm) were under the third percentile. The bilateral testicles were not palpable in the scrotum. Neurological examination showed decreased tendon reflexes, and plantar responses were flexor. Decreased muscle tone in the proximal upper and lower limbs and normal muscle strength were found. Sensation was intact. He had mild ataxic gait with wide-based steps. Nystagmus was noted. During his hospitalization, his hematological examinations showed mild normochromic anemia (Table 1). The examination of urinary organic acids showed significantly increased 2-hydroxyisobutyric acid, glycolic acid, oxalic acid, phosphoric acid, and glyceric acid (Table 1). Interictal electroencephalogram was found with spikes and sharp waves over the bilateral frontal region, anterior midline region, and centrotemporal region during both the waking and sleeping periods (Figure 1). MRI showed lacunar infarction in the left basal ganglia with glial proliferation around and small tiny left transverse sinus, sigmoid sinus, and internal jugular vein (Figure 2). Bilateral cryptorchidism was detected by ultrasonography (Figure 3). Because of the subtle anemia (Hb 113 g/l) without any symptoms, the patient's parents refused to perform the further examination. His blood chemistry test (creatinine, liver enzymes, creatine kinase, lactate, electrolyte, and plasma ammonium), thyroid function (thyroid stimulating hormone and free thyroxine), chest X-ray, and heart ultrasound examination were normal.

**Table 1:** Laboratory results of proband.

	**Proband**	**Normal range**		**Proband**	**Normal range**
Hb (g/dl)	11.3	11–16	2-hydroxyisobutyric acid	3.89	0
MCV (fl)	85.2	79–95	Glycolic acid	8.43	0–2.2
MCH (pg)	28.4	27–31	Oxalic acid	10.43	0
MCHC (g/l)	333	320–360	Phosphoric acid	169.28	0–43
HCT (%)	33.9	35–45	Glyceric acid	7.18	0–0.3

### Gene Analysis

**Figure 4:**
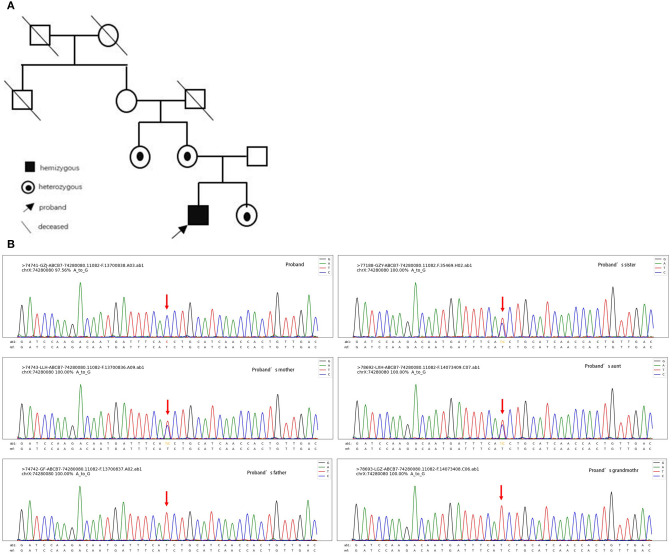
**(A)** Pedigree of the family; **(B)** genetic analysis of the family.

**Figure 5:**
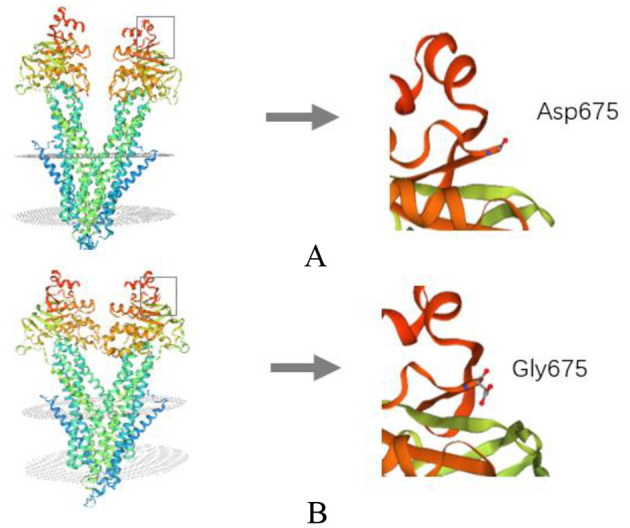
Structure prediction of the mutant protein. **(A)** The wild-type ABCB7 protein structure; **(B)** the mutant protein structure of ABCB7 protein.

Copy number variation sequencing and mitochondrial DNA genome sequencing were normal. Whole exome sequencing revealed a novel missense substitution c.2024A > G (p.D675G) in the ABCB7 gene (NM_004299), which was inherited from his mother (Figure 4). We further tested ABCB7 gene of his younger sister, aunt (mother's sister), and grandmother and found that his sister and aunt were heterozygous (Figure 4). The c.2024A > G mutation was not observed in the gnomAD database and ClinVar database. PolyPhen (http://genetics.bwh.harvard.edu/pph2/), SIFT, and PROVEAN (http://provean.jcvi.org/seq_submit.php) were used to predict putative effect of this mutation on ABCB7 gene. The prediction results of three tools were probably damaging, deleterious, and damaging, respectively (Table 2). According to the ACMG guidelines, the c.2024A > G mutation was classified as likely pathogenic (10). We used SWISS MODEL (https://www.expasy.org/resources/swiss-model) to predict 3D structure of the mutant ABCB7 protein to mimic the effect of the mutated region. As the result presented, the amine acid residue substitution caused an obvious change in local structure compared with wild-type structure (Figure 5). According to the clinical manifestations and gene analysis, we established the diagnosis of XLSA/A.

**Table 2:** Prediction results of the gene mutation.

**Tool**	**Score**	**Reference value**	**Result**
PolyPhen	0.996	0.85–1 probably damaging 0.85–0.15 possibly damaging 0–0.15 benign	Probably damaging
PROVEAN	−6.41	−14–−2.5 deleterious −2.5–14 neutral	Deleterious
SIFT	0.001	0–0.05 damaging 0.05–1 tolerated	Damaging

### Literature Review

So far, there are six families (including this case) reported with XLSA/A due to mutations in ABCB7 gene (Table 3). The total number of these patients who were confirmed mutations in ABCB7 gene was 16. We included P4 and P5 who did not undergo the gene analysis because both of them had typical symptoms and were in the same family of proband P3. Among the six unrelated families, all mutations were missense mutation with one in exon 6, two in exon 9, one in exon 10, one in exon 15, and one in exon 16.

**Table 3:** General information, gene findings, and main symptoms of patients.

**Patient**	**Refernces**	**Gender**	**Age**	Age of symptom onset^*^	**Gene mutation in ABCB7**		**Symptoms/physical signs**												**Other symptoms**
					**Exon**	**Substitution**	**Sit (age)**	**Walk (age)**	**Language (age)**	**Ataxia gait**	**Dysarthria**	**Strabismus**	**Nystagmus**	**Other ataxia symptoms/signs**	**Muscle tone**	**Tendon reflexes**	**Sensory**	**Plantar**	
P1	Hooghe et al. (3)	Male	NA	<2 years	6	c.627A > T (p. E209D)	Normal	4 years, 6 months	Normal	✓	✓	✓	No	Disturbances of coordination in upper or lower limbs	Normal	Normal	Normal	Normal	
P2 (III2)	Hellier et al. and Maguire et al. (5, 18)	Male	52 years	<2 years	9	c.1299G > C (p.V411L)	NA	11 years	NA	✓	✓	✓	✓	Finger–nose ataxia	↑(right side)	NA	Normal	NA	Schizophrenia, walking deteriorated since the age of 40
P3 (III3)		Male	50 years	<2 years	9	c.1299G > C (p.V411L)	NA	3 years	NA	✓	NA	✓	NA	Finger–nose ataxia	NA	Normal	Normal	Downgoing	Walking deteriorated since the age of 45
P4 (II1)		Male	78 years	NA	NA^**^	NA^**^	NA	74 years	NA	NA	NA	NA	NA		NA	NA	NA	NA	Moderate dementia
P5 (II2)		Male	63 years	<2 years	NA^**^	NA^**^	NA	5 years	NA	✓	✓	✓	✓	Finger, nose, and heel–shin ataxia and bilateral dysdiadochokinesia	NA	Brisk	Normal	Flexor	
P6 (IV5)	Pagon et al. and Allikmets et al. (2, 6)	Male	5 years, 6 months	1 year	9	c.1200T > G (p.I400M)	NA	NA	NA	✓	NA	Normal	No	Truncal ataxia, dysmetria, and tremulousness.	Normal	Brisk	NA	Extensor	
P7 (IV12)		Male	2 years, 6 months	6 months	9	c.1200T > G (p.I400M)	Normal	Can't	Normal	–	✓	✓	No		NA	Brisk	Normal	Extensor	
P8 (IV13)		Male	1 year, 11 months	6 months	9	c.1200T > G (p.I400M)	Normal	Can't	Delayed	–	✓	Normal	No	Ataxia while sitting and dysmetria	Normal	Brisk	NA	Extensor	
P9 (III1)		Male	33 years	NA	9	c.1200T > G (p.I400M)	NA	9 years	NA	✓	✓	✓	No	Difficulty with rapid alternating movements, heel-to-shin ataxia, and finger–nose ataxia	NA	Brisk	Normal	Flexor	
P10 (III6)	Protasova et al. (19)	Male	54 years	NA	16	g.742734204C > T (p. G682S)	>10 months	4 years	NA	NA	✓	NA	No		Normal	NA	NA	NA	
P11 (III17)		Male	40 years	NA	16	g.742734204C > T (p. G682S)	NA	7 years	Delayed (3 years)	NA	✓	NA	✓		Normal	NA	NA	NA	
P12 (III18)		Male	38 years	NA	16	g.742734204C > T (p. G682S)	NA	7 years	Delayed (4 years)	NA	✓	NA	✓		Normal	NA	NA	NA	
P13 (III30)		Male	39 years	NA	16	g.742734204C > T (p. G682S)	15 months	4 years	Delayed (4 years)	NA	✓	NA	No		↓	NA	NA	NA	Myoclonus and depression
P14 (IV26)		Male	11 years	NA	16	g.742734204C > T (p. G682S)	14 months	4 years	Delayed (4 years)	NA	✓	NA	✓		Normal	NA	NA	NA	
P15 (IV40)		Male	16 years	NA	16	g.742734204C > T (p. G682S)	6 months	11 months	Delayed (15 months)	NA	✓	NA	✓		↓	NA	NA	NA	
P16 (II1)	Bekri et al. (4)	Male	33 years	<1 year	10	c.1305G > Ap.E433K	4 years	>6 years	Delayed (5 years)	NA	✓	NA	✓	Past pointing and dysdiadochokinesis	NA	NA	NA	NA	Postnatal growth retardation and static intellectual impairment
P17 (II2)		Male	30 years		10	c.1305G > A p.E433K	NA	NA	NA	NA	✓	NA	✓	Past pointing and dysdiadochokinesis	NA	↓	NA	Flexor	
P18	This case	Male	5 years	11 months	15	c.2024A > G p.D675G	1 year	4 years	Delayed (2 years)	✓	✓	Normal	✓		↓	Normal	Normal	Normal	Growth retardation, epilepsy, cryptorchidism, and autism maybe

*NA, not mentioned*;

* *Symptoms include anemia or neurological symptoms*;

** *P4 and P5 did not complete the gene analysis but both of them had typical symptoms and were in the same family as proband P3. They probably had same mutation as P3*.

All patients (18/18) were men. The age of symptom onset reported was no more than 2 years old. The initial symptoms varied like anemia, ataxia, nystagmus, or delayed motor development. Almost all patients except P15 were delayed in walking, and more than half of them could walk without support between 3 and 7 years old. According to the language problem mentioned, eight patients showed delayed language development and 15 patients had dysarthria. Nystagmus (nine out of sixteen patients mentioned in the previous reports, 9/16), dysarthria (15/15), strabismus (5/8), and ataxia gait (7/7) were the most common symptoms. Other symptoms included growth retardation, dementia, intellectual impairment, and impaired gross motor and cognitive development. Two patients had mental disorder: P3 had schizophrenia and P13 was diagnosed with depression. P18 had epilepsy and cryptorchidism. Some patients had changed muscle tone and abnormal tendon reflexes and plantar responses. Evidences for sensory issue were not found in all patients. P2 and P3 were brothers; P2's walking deteriorated since the age of 40, and P3's walking deteriorated since the age of 45.

Twelve affected patients and seven patients' mothers underwent the hematological investigations (Table 4). Hemoglobin of 4/12 patients and 6/7 carriers was in normal range. Eight of 12 patients and 1/7 carriers had mild anemia, and most of them were microcytic hypochromic anemia with nookrmal range of serum iron, total iron binding capacity, and transferrin saturation. Erythrocyte protoporphyrin of 8/8 patients and 4/6 carriers was significantly higher than the normal range. Microcytic, hypochromic red cells, abnormally shaped cells, pappenheimer bodies, and anisocytosis were commonly seen in blood films. Bone marrow examination were performed in four patients and two carriers, and all of them had ringed sideroblasts. P9 received treatment with oral vitamin B6, although it did not alter hematocrit (HCT), mean cell volume (MCV), mean cell hemoglobin (MCH), or reticulocyte count. P16 received pyridoxine supplementation, but the hemoglobin deficiency was refractory. No one needed blood transfusion. P1, P7, and P9 underwent cerebral imaging at the age of 4, 2.5, and 33, respectively, and no abnormalities were found. Brain MRI were performed in P2 and P3 at the age of 50 and showed atrophic cerebellum, and P3 had atrophy of the pons and medulla. P16 underwent computed tomography of the brain at age 18, and striking, selective cerebellar hypoplasia was found. In this case, brain MRI showed lacunar infarction in the left basal ganglia with glial proliferation around and small tiny left transverse sinus, sigmoid sinus, and internal jugular vein, but his cerebellum structure was normal.

**Table 4:** Hematological findings.

**Patient**	**Hemoglobin (g/dl)**	**Hematocrit (%)**	**Mean cell volume (fl)**	**Mean cell hemoglobin (pg)**	**Serum iron**	**Total iron binding capacity**	**Transferrin saturation**	**Transferrin**	**Ferritin**	**Erythrocyte protoporphyrin**	**Ringed sideroblasts in bone marrow**	**Blood film**
P1^*^	11.6 (10.0–13.0)	35.7 (30–40)	63.6 (78–100)	20.7 (28–34)	79 μg/dl (29–91)	380 μg/dl (250–420)	21	338 mg/dl (200–360)	23.3 μg/l (10–92)	Total 4,656 mg/l RBC (200–500)	Present (5%−10%)	Microcytic, hypochromic red cells with anisocytosis, abnormally shaped cells and pappenheimer bodies
P2	12.1 (13–17)	36 (39–50)	84 (78–98)	26 (27–34)	20 μmol/l (14–33)	39 μmol/l (45–75)	51	NA	95 μg/l (14–200)	Free 3.1 μmol/l (0.4–1.7)	NA	Anisocytosis, poikilocytosis, target cells, Howell-Jolly bodies, and pappenheimer bodies
P3*	14.3 (13–17)	42 (39–50)	77 (78–98)	24 (27–34)	30 μmol/l (14–33)	67 μmol/l (45–75)	45	NA	125 μg/l (14–200)	Free 4.9 μmol/l (0.4–1.7)	Present	Hypochromic red cells and pappenheimer bodies
P4	10.2 (13–17)	29 (39–50)	83 (78–98)	NA	NA	NA	NA	NA	NA	NA	NA	
P5	13.5 (13–17)	41 (39–50)	74 (78–98)	NA	NA	NA	NA	NA	38 μg/l (14–200)	NA	NA	
P6^*^	Anemia^†^	30	68	NA	73 μg/dl (55–155)	262 μg/dl (300–350)	28 (20–50)	NA	17 ng/ml (20–160)	Free 16.8 μg/g haem (1–5 μg/g haem)	Present	Microcytic, hypochromic red cells with anisocytosis and cigar-shaped cells
P7	Anemia^†^	30	58	NA	NA	NA	NA	NA	NA	Free 140 mol/mol haem (8–18)	NA	Dimorphic red cell population
P8	Anemia^†^	29	58	NA	NA	NA	NA	NA	NA	NA	NA	Dimorphic red cell population
P9	Anemia^†^	35	67	NA	103 μg/dl (55–155)	314 μg/dl (300–350)	33	NA	160 ng/ml (20–160)	184 mol/mol haem (8–18)	Present (80%)	Microcytic, hypochromic red cells with marked poikilocytosis, shift to the left, and heavy stippling
P16^*^	10–10.8 (13.5–17.5)		61.8–62.2 (80–100)	18.7–20.0 (27–32)	7 μmol/l (10–40)	45 μmol/l (45–77)	16 (15–30)	NA	156–277 μg/l (15–300)	Total 25.7 μmol/l (0.4–1.7)	NA	
P17	8.4–9.9 (13.5–17.5)		58.7–60.2 (80–100)	16.8–19.6 (27–32)	7 μmol/l (10–40)	41 μmol/l (45–77)	17 (15–30)	NA	277–331 μg/l (15–300)	Total 19.5 μmol/l (0.4–1.7)	NA	
P1's mother	12 (11.7–16.0)	38 (35–47)	83.8 (81–100)	26.6 (27–34)	112 μg/dl (37–145)	300 μg/dl (228–428)	37	247 mg/dl (200–360)	69.9 μg/l (13–400)	Total 3,826 mg/l RBC (200–550)	NA	Pappenheimer bodies
P3's mother	13.1 (13–17)	NA	93 (78–98)	30 (27–34)	19 μmol/l (14–33)	63 μmol/l (45–75)	30	NA	135 μg/l (14–200)	Free 1.4 μmol/l (0.4–1.7)	NA	Pappenheimer bodies
P6's mother	NA	40	90	NA	71 μg/dl (55–155)	329 μg/dl (300–350)	22 (20–50)	NA	40 ng/ml (20–160)	Free 27 mol/mol haem (8–18)	Present	Dimorphic red cell population
P7's mother	NA	39	84	NA	63 μg/dl (55–155)	314 μg/dl (300–350)	20 (20–50)	NA	62 ng/ml (20–160)	Free 10 mol/mol haem (8–18)	NA	Normal
P8's mother	NA	37	83	NA	72 μg/dl (55–155)	278 μg/dl (300–350)	26 (20–50)	NA	42 ng/ml (20–160)	18 mol/mol haem (8–18)	NA	Dimorphic red cell population
P9's mother	NA	37	83	NA	82 μg/dl (55–155)	278 μg/dl (300–350)	29 (20–50)	NA	88 ng/ml (20–160)	62 mol/mol haem (8–18)	Present	Dimorphic red cell population
P16's mother	11.2–13.4 (12.5–16.5)	NA	80.9–81.3 (80–100)	26.9–27 (27–32)	9 μmol/l (10–40)	75 μmol/l (45–77)	12 (15–30)	NA	76–105 μg/l (15–300)	NA	NA	
P18	11.3 (11–16)	33.9 (35–45)	85.2 (79–95)	28.4 (27–31)	NA	NA	NA	NA	NA	NA	NA	

*NA, not mentioned*;

* *proband*;

† *anemia was diagnosed, but data was not mentioned*.

## Discussion

All patients presented here were male, consistent with the X-linked feature. According to our patient's genealogy, the same missense variant of ABCB7 gene was detected in his grandmother's two daughters, but genetic testing of her peripheral blood samples was normal. Moreover, proband's grandfather who died several years ago did not have any neurological symptoms. The proband's grandmother was very likely to harbor germline mosaicism for the ABCB7 gene variant. Similar cases were also reported by Pianese et al. (11) and Okamoto et al. (12). However, it is difficult to evaluate germinal mosaicism, because it is complicated to derive eggs from the ovaries if a maternally originated mutation is suspected. In addition, because of the identified mutation in the offspring (patient's aunts) from apparently unaffected parents (patient's grandmother), germline mosaicism should be suspected (13).

Symptoms of XLSA/A usually appeared within the first 2 years of life. All patients showed delay in motor development. Early onset of ataxia, dysarthria, nystagmus, strabismus, and ataxia gait were the most common symptoms. Growth retardation, mental disorder, and intellectual impairment were also found in the patients. In this case, the patient was diagnosed with epilepsy in infancy and had cryptorchidism, which had not been described in other cases. Most patients had mild sideroblastic anemia with obviously increased erythrocyte protoporphyrin. Seven carriers did not show neurologic abnormality. The red cells from the obligatory carriers were affected to a variable extent. Most of the carriers showed raised protoporphyrin levels and some had ringed sideroblasts in the bone marrow. In this case, several abnormal urinary organic acids were detected; 2-hydroxyisobutyric acid is an essential endogenous metabolite and is reported to be increased in kidney disease, diabetes mellitus, and myocardial infarction (14). Upregulation of glycolic acid and oxalic acid was reported to be strongly associated with primary hyperoxaluria and kidney stones. Increased phosphoric acid is also related to kidney stones and chronic kidney disease. Glyceric acid is elevated in genetic disorders (hyperoxaluria type I and II). There is no evidence for an association between the diseases mentioned above and the abnormal findings presented in this patient. However, follow-up visit was necessary to track whether this patient appeals the early clinical manifestations of other metabolic disorders. Cerebral imaging of some patients showed atrophic cerebellum. It is hypothesized that these various symptoms may be dependent on the different location of gene mutations and different ages. According to the limited data, patients (P16 and P17) with mutations in exon 10 had more severe anemia than patients with mutations in other exons. Two families had mutations in exon 9, and P7 and P9 from the same family who underwent cerebral imaging at age 2.5 and 33, respectively, had normal structure. However, brain MRI of P2 and P3 performed at their fifties showed atrophic cerebellum. Moreover, brain MRI of P1 and P18 at their early age also showed that their cerebellums were normal. Cerebellum atrophy probably occurs in the later stages of the disease and this may be related to walking deterioration. Considering the limited number of cases reported, our speculation needs to be confirmed on a larger number of patients.

**Figure 6:**
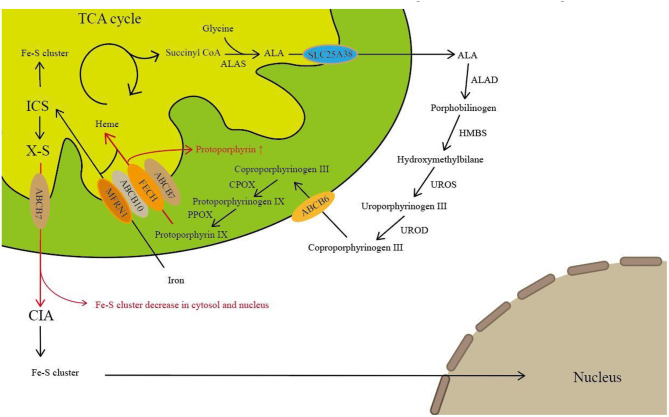
ABCB7 plays a role in heme metabolism and Fe–S cluster transfer. The heme biosynthesis begins with the condensation of glycine and succinyl-CoA to form ALA by ALAS in the mitochondrial matrix. Then, ALA is exported to the cytosol where it is converted to coproporphyrinogen III by following four enzymatic steps. Subsequently, coproporphyrinogen III is transferred into intermembrane space of the mitochondria by ABCB6 and converted to protoporphyrin IX through the next two enzymatic steps. In the last step of heme biosynthesis, iron is incorporated into protoporphyrin ring catalyzed by FECH, which interacts directly with ABCB7 and ABCB10. Iron delivery into the mitochondrial matrix by MFRN. Mitochondrial iron is used to generate Fe–S cluster. A so far uncharacterized compound (X) is exported via ABCB7. In the cytoplasm, the CIA machinery inserts Fe–S cluster into cytosolic and nuclear Fe–S proteins. The hypothetical effects of defects in ABCB7 are presented in red arrow. ALA, 5-aminolevulinic acid; ALAS, α-aminolevulinate synthase; MFRN, iron importer mitoferrin; FECH, ferrochelatase; CIA, cytosolic Fe–S cluster assembly machinery.

The present study has demonstrated the involvement of ABCB7 in the biosynthesis of heme via interaction with ferrochelatase and ABCB10 (8, 9). The heme biosynthetic pathway involves eight enzymatic reactions, beginning with the condensation of glycine and succinyl-CoA to form a 5-aminolevulinic acid (ALA) by α-aminolevulinate synthase (ALAS) in the mitochondrial matrix. Through the following steps, protoporphyrin IX was formed in the intermembrane space of the mitochondria. In the last step of heme biosynthesis, iron is inserted into the protoporphyrin ring by ferrochelatase (FECH) (Figure 6). During the impaired ABCB7 activity, iron remains trapped in the mitochondria and the levels of Fe–S cluster-dependent enzyme activities are declined in affected cells, resulting in the compromised ALAS2 translation by binding of IRPs to the IRE located in the 5′-UTR of ALAS2 mRNA (15). In addition, studies found that FECH could interact with ABCB7 and ABCB10 in heme synthesis (8, 9). Dysfunction of ABCB7 may affect both the first step and last step of heme synthesis. In line with previous reports, patients with ABCB7 mutation always have sideroblastic anemia with increased level of protoporphyrin, and it is seemed that ABCB7 may have affected the last step of heme biosynthesis more than the first step.

So far, the molecular mechanisms linking ABCB7 mutations to ataxia are poorly understood. Knockdown of ABCB7 leads to dysfunction of cytosolic Fe–S proteins, which was identified both *in vivo* and *in vitro* (8, 16). Defects in ABCB7 affect its transfer function that exports a sulfur-containing compound into the cytosol (Figure 6). Reduction of cytosolic Fe–S proteins may result in the alteration of a plethora of cellular mechanisms such as altered DNA repair, glycolysis, fatty acid synthesis, purine catabolism, and ribosome biogenesis (17). Additionally, the ABCB7 gene is highly expressed not only in the bone marrow but also in the cerebellum, which may be thought to be the reason to induce ataxia in patients with ABCB7 deficiency. However, in order to fully understand the particular role and the extent to which extramitochondrial Fe–S clusters contribute to this or other human diseases, further investigations are required.

## Conclusion

Patients with XLSA/A always present striking ataxia and mild anemia at the early age. Most patients have slightly abnormal hemoglobin without obvious symptoms of anemia, which could be easy to misdiagnose at an early stage. Bone marrow examination, protoporphyrin, and genetic test should be considered in male patients with early-onset ataxia.

## Ethics Statement

Written informed consent was obtained from the individuals for the publication of any potentially identifiable images or data included in this article.

## Author Contributions

SX and YJ were residents and wrote the paper. SL and JX were the attending physicians. DM and LL were professors, and they all participated in the care of the patient. PH was a resident and performed the electroencephalogram. QH analyzed and interpreted the patient's data. All authors contributed to the article and approved the submitted version.

## Conflict of Interest

The authors declare that the research was conducted in the absence of any commercial or financial relationships that could be construed as a potential conflict of interest.
